# Beneficial association of serum ghrelin and peptide YY with bone mineral density in the Newfoundland population

**DOI:** 10.1186/1472-6823-13-35

**Published:** 2013-09-23

**Authors:** Peyvand Amini, Farrell Cahill, Danny Wadden, Yunqi Ji, Pardis Pedram, Sangeetha Vidyasankar, Yanqing Yi, Wayne Gulliver, Gary Paterno, Hongwei Zhang, Alecia Rideout, Guang Sun

**Affiliations:** 1 Division of Medicine, Faculty of Medicine, Memorial University, 300 Prince Philip Drive, St. John’s, NF, Canada

**Keywords:** Ghrelin, Peptide YY, Osteoporosis, Bone density

## Abstract

**Background:**

Ghrelin and peptide YY (PYY) are appetite regulating hormones secreted from the gastrointestinal tract (gut). Aside from their known effect on energy homeostasis, accumulating data indicates that these gut hormones also affect bone metabolism. However, data regarding the influence of ghrelin and PYY on bone density in humans is very limited, and the results are inconclusive. Therefore, this study was designed to investigate the potential association between circulating ghrelin and PYY with bone density indices in the general population.

**Methods:**

A total of 2257 adult subjects from the CODING (Complex Diseases in the Newfoundland Population: Environment and Genetics) study participated in this investigation. Acylated ghrelin and total PYY were measured in serum after a 12-hour fasting, with the Enzyme- Linked Immunosorbent Assay (ELISA) method. Bone mineral density was measured by dual-energy X-ray absorptiometry at the spine, femoral neck, and total hip. Multiple regression analyses adjusting for age, BMI, physical activity, smoking, and alcohol consumption were employed to analyze the association between serum ghrelin and PYY with bone mineral density parameters.

**Results:**

Significant positive associations of ghrelin concentration with L2-L4 BMD, L2-L4 Z-score, femoral neck BMD, femoral neck Z-score, total hip BMD, and total hip Z-score were found in women. No significant correlations between ghrelin and bone density indices were present in men. After dividing the female group into pre-menopausal and post-menopausal, ghrelin was positively correlated with femoral neck Z-score, and total hip Z-score in pre-menopausal women and L2-L4 BMD, and Z-score in post-menopausal group. Moreover, no significant association was discovered between serum PYY and bone density at any site.

**Conclusion:**

Our results suggest a beneficial association of circulating ghrelin concentration with bone density in women at the population level. This association is independent of major confounding factors including BMI, physical activity, age, alcohol consumption, and smoking. Effect of menopause on this association seemed to be site specific. However, PYY does not seem to be associated with bone density parameters.

## Background

Osteoporosis is a global problem. According to the International Osteoporosis Foundation (IOF) data, the annual treatment cost for osteoporosis fractures of people in the workplace in the USA, Canada and Europe is almost 48 billion USD [1]. Therefore, understanding the potential factors that cause osteoporosis is of great value. Most cases of osteoporosis are idiopathic because of estrogen deprivation and aging [2]. However, many other factors are involved in the pathogenesis of osteoporosis. In populations aged 50 years and over, secondary causes of osteoporosis such as endocrine, gastrointestinal, and connective tissue diseases, have been found in 41.4% of women and 51.3% of men [3]. In addition, the gastrointestinal hormones, ghrelin and PYY, which aid in energy homeostasis and weight management, have been found to be involved in the regulation of bone density [4,5]. Ghrelin is a 28 amino acid appetite stimulant peptide secreted primarily from the stomach, and PYY is an appetite suppressant hormone secreted from the enteroendocrine cells of the ileum and colon [6-8].

The initial investigations regarding the effect of ghrelin on bone density, that were performed on rodents and in vitro studies, have shown that ghrelin increases osteoblast replication, osteoblast specific gene expression, differentiation of osteoblast markers, and bone mineral density (BMD) [4,9,10]. Human studies regarding the effect of ghrelin on bone density are very limited and the results are inconsistent. In a study with 137 elderly men, ghrelin was positively correlated with femoral neck BMD [11]. In another study, eleven months after gasterectomized surgery, a significant decrease in circulating ghrelin and bone mineral density was found [12]. However, no association was found between serum ghrelin concentration with femoral neck BMD or lumbar spine BMD in 81 Korean men [13]. A study by Makovey et al. also did not find any significant correlation between ghrelin concentration and bone mass parameters in 79 pairs of opposite sex twins [14]. Similarly, Weiss et al. did not find any association between ghrelin and BMD in older men or women after adjusting for age and BMI [15].

Results from animal studies on the effect of PYY on bone density are also inconsistent. The hypothalamic Y2 receptor serves as the receptor of PYY. Y2 receptor deficient mice have increased trabecular bone volume, and rate of bone mineralization and formation [5]. However, PYY deficient mice developed a decrease in trabecular bone mass and osteopenia [16]. Human studies on the effect of PYY on bone density are extremely limited in terms of a general population level, as previous studies have only been performed on special groups such as anorexic patients or women experiencing exercise [17-20].

Emerging data suggest the functionally related gut hormones, ghrelin and PYY, are linked to bone metabolism and BMD. However, data from humans are limited, the results are contradictory and subject to statistical errors due to small sample size. Also, BMD is a complex physiological measure and many factors can exert a significant effect on it. Therefore, it is important to evaluate whether the possible associations between these two important gut hormones and bone mineral density are independent of major confounding factors. The objectives of the current study were: 1) to determine if ghrelin and PYY are associated with bone density parameters in a large population-based cohort; 2) to evaluate whether this possible association is different in men and women, and also in pre- and post-menopausal women; and 3) to explore whether the possible associations between ghrelin or PYY and bone mineral density are independent of age, BMI, physical activity, alcohol consumption, and smoking.

## Methods

### Study population

A total of 2,257 subjects from the CODING (Complex Diseases in the Newfoundland population: Environment and Genetics) study, including 551 men and 1706 women were recruited in the present study through advertisement in public media and word of mouth by previous volunteers. All volunteers were at least third-generation Newfoundlander, between the ages of 20 and 79 years old, without any serious metabolic, cardiovascular, or endocrine diseases, and women were not pregnant at the time of the study.

### Ethical considerations

This study was approved by the Health Research Ethics Authority of the Faculty of Medicine of Memorial University, St. John’s, Newfoundland, Canada. Informed assent and consent were obtained from all of the volunteers.

### Anthropometric measurements

Anthropometric measurements were performed with participants dressed in a standardized hospital gown. Standing height was measured to the nearest 0.1 cm using a fixed stadiometer. Subjects were weighed to the nearest 0.1 kg using a platform manual scale balance (Health O Meter, Bridgeview, IL). BMI was calculated from weight and height in kilograms per square meter [(weight-kg)/(height-m)^2^].

### Body composition and bone mineral density measurements

The measurements of bone mineral mass were carried out by dual-energy X-ray absorptiometry (DXA) Lunar Prodigy (GE Medical Systems, Madison, WI) equipped with encore software v12.3. Volunteers were scanned by the same technician in standardized clothing (hospital gown) with no removable metal objects, while lying flat on their backs with arms at their sides. In all subjects, BMD was measured at the sites of lumbar spine, femoral neck, and total hip. Moreover, Z-score and T-score were measured for these areas.

According to the World Health Organization (WHO), T-score ≥ -1 is considered normal, T-score < -1 and > -2.5 is considered osteopenia, and T-score ≤ -2.5 is considered osteoporosis [21].

### Physical activity

The Baecke questionnaire was used for evaluation of the subject’s physical activity based on the work, sports, and leisure activity [22].

### Blood analysis

Venous blood samples were obtained from all volunteers in the morning after an overnight fast (12 hours). Serum samples were isolated from blood and stored at −80°C until assayed.

Serum acylated ghrelin was measured with an Enzyme - Linked Immunosorbent Assay (ELISA) method (Human Acylated Ghrelin Enzyme Immunoassay Kit of Spibio-bertin pharma). Acylated ghrelin is unstable and sensitive to de-acylation. Therefore, all samples used for the measurement of acylated ghrelin were thawed for the first time on the day of analysis, and while running ELSIA kits, all work was completed on ice. Intra- and inter-assay coefficients of variation (CV) were 5.7% and 17% respectively.

Serum total PYY concentration was measured with the ELISA kit from Millipore (Millipore Corporation Pharamaceuticals, Billerica, MA, USA). The intra-assay CV was 4.8% - 5.4% and inter-assay CV was 5.1% [23].

### Statistical analysis

Statistical analyses were performed using SPSS, version 20.0 (SPSS Inc, Chicago). All tests were two-sided and p value < 0.05 was considered to be statistically significant.

Evaluation of data normality was performed with the Kolmograv- Smirnov test. Demographic and physical characteristics values were expressed as mean (standard deviation). Logarithmic transformation was performed for ghrelin, PYY and bone density parameters, except Z-score (because of the negative values) that were not normally distributed. These values were reported as median, minimum and maximum in the results. Analyses were performed on the entire cohort and, as well, on men and women separately. Women were further subdivided according to their menopausal status and the analyses were conducted between pre- and post-menopausal groups. Pearson correlation was used to determine the relationship between ghrelin and PYY and bone mineral density indices. Stepwise multiple regression analyses were used to identify predictors of bone density indices. Gut hormones and other identified confounders of bone density such as age, BMI, physical activity, smoking, and alcohol consumption, were considered independent variables. Percentage of body fat as the more accurate measure for body composition was also replaced with BMI to see whether the effect of body fat percentage differed from BMI. The results were similar. Therefore, in order to remain consistent with previous literature, BMI was entered into the model.

## Results

### Subject characteristics

Mean and standard deviation of demographic and physical characteristics of the subjects are presented in Table 1. Ghrelin, PYY, and bone density parameters are described as median, minimum and maximum in Table 2. According to the WHO criteria (based on the L2-L4 T-score), 80.8% of volunteers had normal bone density, 16.9% were osteopenic, and 2.2% were osteoporotic. According to the femoral neck T-score, 76.6% were normal, 22.6% and 0.7% met the criteria of osteopenia and osteoporosis respectively, and based on total hip T-score 83.5%, 16.2%, and 0.3% were normal, osteopenic, and osteoporotic respectively.

**Table 1:** Demographic and physical characteristics of volunteers

	**Entire cohort**	**Female**	**Male**
	**(n = 2257)**	**(n = 1706)**	**(n = 551)**
	**Mean (SD)**	**Mean (SD)**	**Mean (SD)**
**Age (yr)**	43.1 (12.3)	44 (11.7)	40.3 (13.7)
**Weight (kg)**	73.4 (15.8)	69.5 (14.1)	85.4 (14.7)
**Height (cm)**	165.6 (8.5)	162.3 (5.9)	176.1 (6.5)
**BMI (kg/m**^**2**^**)**	26.7 (5.1)	26.4 (5.2)	27.5 (4.5)
**Percent body fat (%)**	34.5 (9.4)	37.5 (7.6)	25.1 (7.8)
**Percent trunk fat (%)**	36.6 (9.7)	38.8 (8.8)	30 (9.3)
**Percent android fat (%)**	41.9 (11.3)	43.7 (10.6)	36.1 (11.4)
**Percent gynoid fat (%)**	40.8 (9.8)	44.7 (6.6)	28.6 (8)
**Total fat mass (kg)**	25.5 (10.3)	26.6 (10.2)	22.1 (9.8)
**Total lean mass (kg)**	44.6 (10.6)	39.7 (53.9)	59.9 (7.9)

**Table 2:** Descriptive statistics for ghrelin, PYY, and bone density indices

**Variables**	**Entire cohort**		**Female**		**Male**	
	**Median**	**Min-Max**	**Median**	**Min-Max**	**Median**	**Min-Max**
**Ghrelin (pg/ml)**	194.7	0.74–2329.09	193.44	0.74–2329.09	196.73	2.12–2289.26
**PYY (pg/ml)**	95	3.67–368.53	92.52	3.67–368.53	103.67	8.37–364.66
**Spine BMD (g/cm**^**2**^**)**	1.21	0.76–1.85	1.20	0.76–1.85	1.26	0.81–1.78
**Left Hip BMD (g/cm**^**2**^**)**	0.98	0.52–1.83	0.96	0.52–1.65	1.04	0.67–1.83
**Total hip BMD (g/cm**^**2**^**)**	1.02	0.61–1.68	0.99	0.61–1.58	1.1	0.76–1.68
**L2-L4 Z score (%)**	0.2	−4.1–6.1	0.2	−4.1–6.1	0.1	−3.4–4.8
**Femur Neck Z score (%)**	0.1	−2.5–5.7	0.2	−2.5–5	0.1	−2–5.7
**Total hip Z-score (%)**	0.2	−3.12–4.4	0.21	−3.12–4.12	0.18	−2.63–4.4

### Pearson correlation of ghrelin and PYY with bone density measures

Pearson correlation analyses showed positive correlations between ghrelin and L2-L4 Z-score, femoral neck Z-score, and total hip Z-score in the entire cohort (r = 0.05, p = 0.03, r = 0.07, p = 0.004, and r = 0.05, p = 0.03 respectively), and in females (r = 0.08, p = 0.006, r = 0.09, p = 0.003, and r = 0.07, p = 0.006 respectively).

For PYY, there was no significant association with any of the bone density parameters either in the entire cohort or in the males and females separately.

### Multiple regression analyses of ghrelin and PYY with bone density indices adjusting for BMI, age, physical activity, smoking and alcohol consumption

Stepwise multiple regression analyses were performed to clarify the determinants of BMD and Z-score in males and females separately. In females, there were significant positive associations between ghrelin and L2-L4 BMD and Z-score, femoral neck BMD and Z-score, and total hip BMD and Z-score (Table 3). [For Z-score, age was not included in the model because Z-score is the number of standard deviations above or below what is normally expected for someone of their age, sex, and ethnic or racial origin].

**Table 3:** **Regression analyses of ghrelin with BMD and Z-scores in women and men**^**1**^

	**Female**					**Male**				
	**Variables**	**β**^**∗**^	**(95% CI)**^**✝**^	**P**	**R**^**2**^	**Variables**	**β**	**(95% CI)**	**P**	**R**^**2**^
**L2-L4 BMD**	Age	−0.003	(−0.003, −0.002)	<0.001	0.1	BMI	0.006	(0.003, 0.009)	<0.001	0.045
	BMI	0.005	(0.004, 0.007)	<0.001		Age	−0.001	(−0.002, 0.000)	0.012	
	Smoking	−0.043	(−0.065, −0.021)	<0.001						
	Ghrelin	0.009	(0.002, 0.017)	0.015						
**Femoral Neck BMD**	Age	−0.004	(−0.005, −0.004)	<0.001	0.238	Age	−.006	(−0.006, −0.005)	<0.001	0.361
	BMI	0.008	(0.006, 0.009)	<0.001		BMI	.012	(0.010, 0.015)	<0.001	
	PA^2^	0.011	(0.007, 0.016)	<0.001		PA	.015	(0.007, 0.023)	<0.001	
	Smoking	−0.044	(−0.065, −0.023)	<0.001		Smoking	−.047	(−0.083, −0.010)	0.012	
	Ghrelin	0.008	(0.000, 0.015)	0.04		Alcohol	.001	(0.000, 0.002)	0.020	
**Total Hip BMD**	BMI	0.011	(0.009, 0.012)	<0.001	0.234	BMI	.013	(0.011, 0.016)	<0.001	0.247
	Age	−0.003	(−0.003, −0.002)	<0.001		Age	−.002	(−0.003, −0.001)	<0.001	
	PA	0.009	(0.005, 0.014)	<0.001		PA	.013	(0.005, 0.020)	0.002	
	Smoking	−0.036	(−0.057, −0.016)	<0.001		Alcohol	.001	(0.000, 0.002)	0.019	
	Ghrelin	0.009	(0.002, 0.016)	0.009		Smoking	−.037	(−0.072, −0.002)	0.036	
**L2-L4 Z-score**	Smoking	−0.458	(−0.675, −0.241)	<0.001	0.022					
	Ghrelin	0.102	(0.027, 0.177)	0.007						
**Femoral Neck Z-score**	Smoking	−0.338	(−0.502, −0.173)	<0.001	0.04	PA	0.158	(0.092, 0.224)	<0.001	0.085
	PA	0.078	(0.044, 0.113)	<0.001		BMI	0.055	(0.031, 0.079)	<0.001	
	Ghrelin	0.073	(0.015, 0.131)	0.013						
	BMI	0.013	(0.003, 0.024)	0.015						
**Total Hip Z-score**	BMI	0.043	(0.033, 0.053)	<0.001	0.076	BMI	0.08	(0.058, 0.102)	<0.001	0.137
	Smoking	−0.286	(−0.447, −0.125)	0.001		PA	0.122	(0.059, 0.185)	<0.001	
	PA	0.059	(0.026, 0.093)	0.001		Alcohol	0.007	(0.000, 0.015)	0.042	
	Ghrelin	0.081	(0.024, 0.137)	0.005						

^1^Regression model adjusted for age, BMI, alcohol consumption, physical activity, and smoking^2^ Physical Activity^*^ Unstandardized β coefficients ^✝^95% Confidence Interval.

For PYY after entering the variables into the model, no significant association was found between PYY and BMD or Z-score values.

### Influence of menopause on the relationship between ghrelin and bone density

To evaluate the influence of menopause on the association between ghrelin and bone density, multiple regression analyses were performed in females after they were divided into pre- and post-menopausal groups. Significant associations were seen between ghrelin and femoral neck and total hip Z-scores in pre-menopausal women. In post-menopausal group ghrelin was positively associated with L2-L4 BMD, and Z-score (Table 4).

**Table 4:** **Regression analyses of ghrelin with BMD and Z-scores in women based on menopausal status**^**1**^

	**Pre-menopausal (N = 971)**					**Post-menopausal (N = 653)**				
	**Variables**	β^*^	**(95% CI)**^**✝**^	**P**	**R**^**2**^	**Variables**	**β**	**(95% CI)**	**P**	**R**^**2**^
**L2-L4 BMD**	BMI	0.005	(0.003, 0.006)	<0.001	0.05	BMI	0.006	(0.003, 0.008)	<0.001	0.088
	Smoking	−0.032	(−0.059, −0.004)	0.023		Smoking	−0.055	(−0.092, −0.017)	0.005	
						Age	−0.002	(−0.004, −0.001)	0.001	
						Ghrelin	0.014	(0.001, 0.027)	0.037	
**Femoral Neck BMD**	BMI	0.008	(0.006, 0.010)	<0.001	0.139	Age	−0.004	(−0.006, −0.003)	<0.001	0.195
	Age	−0.003	(−0.004, −0.002)	<0.001		BMI	0.007	(0.005, 0.009)	<0.001	
	PA^2^	0.013	(0.007, 0.019)	<0.001		Smoking	−0.044	(−0.077, −0.012)	.007	
	Smoking	−0.039	(−0.067, −0.010)	0.007		PA	0.008	(0.001, 0.015)	.019	
**Total Hip BMD**	BMI	0.01	(0.008, 0.011)	<0.001	0.164	BMI	0.011	(0.009, 0.013)	<0.001	0.263
	PA	0.014	(0.008, 0.019)	<0.001		Age	−0.003	(−0.004, −0.002)	<0.001	
	Smoking	−0.034	(−0.063, −0.006)	0.017		Smoking	−0.041	(−0.071, −0.011)	0.008	
**L2-L4 Z-score**	Smoking	−0.394	(−0.673, −0.116)	0.006	0.012	Smoking	−0.596	(−0.964, −0.229)	0.002	0.034
						Ghrelin	0.135	(0.006, 0.263)	0.04	
**Femoral Neck Z-score**	PA	0.106	(0.058, 0.154)	<0.001	0.051	Smoking	−0.421	(−0.658, −0.185)	.001	0.025
	Smoking	−0.304	(−0.538, −0.071)	0.011						
	BMI	0.018	(0.003, 0.033)	0.018						
	Ghrelin	0.096	(0.017, 0.175)	0.018						
**Total Hip Z-score**	BMI	0.043	(0.028, 0.058)	<0.001	0.083	BMI	0.043	(0.029, 0.057)	<0.001	0.084
	PA	0.092	(0.044, 0.140)	<0.001		Smoking	−0.32	(−0.552, −0.089)	0.007	
	Smoking	−0.315	(−0.546, −0.085)	0.007						
	Ghrelin	0.107	(0.029, 0.185)	0.008						

^1^Regression model adjusted for age, BMI, alcohol consumption, physical activity, and smoking^2^ Physical Activity^*^ Unstandardized β coefficients^✝^ 95% Confidence Interval.

## Discussion

In the present study, we examined the associations between the levels of circulating ghrelin and PYY, with bone mineral density indices controlling for major confounding factors in the Newfoundland population. The most important finding from our study is that circulating fasting ghrelin concentration is significantly and positively correlated with femoral neck, total hip, and lumbar spine bone mineral densities and Z-scores in females. More importantly, we demonstrated that the association of ghrelin with bone mineral density is independent of age, body composition, alcohol consumption, physical activity, and smoking. We found out that serum PYY is not significantly correlated with any of the bone density measures in this study. To our knowledge this is the largest human study that simultaneously evaluated the relationship of the two gut hormones, ghrelin and PYY, with bone mineral density with comprehensive control of major confounding factors.

Similarly, a study with the sample size of 137 men aged 55 years or older revealed a positive correlation between ghrelin and femoral neck BMD [11]. The average ages in both women and men in our study are younger, and the results are very reliable with such a large sample size.

Data from both cultured cell based and animal experiments supported the significant association between ghrelin and BMD. An animal study has shown that ghrelin receptors are present in osteoblasts and ghrelin can increase osteoblast proliferation and differentiation markers [4]. Moreover, gasterectomy in mice, in which ghrelin secretion is significantly reduced, can cause decreased bone density [24].

The mechanism by which ghrelin increases bone mineral density has yet to be completely understood. Ghrelin is a natural ligand for the growth hormone secretagogue receptor, and growth hormone increases bone density. Therefore, ghrelin may also affect bone density through the growth hormone related pathway. Moreover, previous studies have shown that osteoblasts express the ghrelin receptor. Ghrelin stimulates both osteoblast cell proliferation and differentiation [4]. However, Delhanty et al. did not find expression of GHS-R1a (Growth hormone secretagogue Receptor-1a) in osteoblasts. They found out that the effect of ghrelin on bone density is through ERK and PI3K, and MAPK pathway [25]. A recent study on wild type and ghrelin receptor deficient mice has shown that ghrelin can inhibit osteoclastogenesis and this effect is age dependent. With aging inhibitory effect of ghrelin on osteoclasts increases [26].

Data from some studies do not support the association between ghrelin and bone density. In ghrelin knockout mice, bone mineral density and bone mineral content between ghrelin −/− mice and wild type mice were similar [27]. There was no significant association between ghrelin and BMD in a study consisting of 80 male adults. In this study, the effect of alcohol, smoking, and physical activity was not controlled [13]. Also, in a study with 977 old adults, no significant association was found between ghrelin and BMD in either sex after controlling for age and BMI [15]. Ghrelin has extremely high standard deviation, and therefore studies on ghrelin need a very large sample size to have reasonable statistical power. Otherwise it is likely to have type II error. Caution should be taken on either negative or positive results from the studies with small sample sizes.

In our study, the positive association between ghrelin and BMD was found only in females. The reason for the sex difference in the association between ghrelin and BMD is unknown, and could be due to the relatively smaller number of male subjects, differences in sex hormones, or other unknown factors.

Bone density is a complex physiological marker. Many factors can potentially be involved in the regulation of BMD. In the present study, one of the important goals was to clarify if the significant positive association of ghrelin with bone mineral density is secondary to any confounding factor. We were able to demonstrate that the positive association is indeed independent of the major confounding factors available in the study.

Physical activity and age are important in determining bone density [28,29]. In our study, ghrelin was positively correlated with age. Previous primary studies have shown that bone density decreases at most sites after age of fifty due to trabecularization of cortical bones [30,31]. Physical activity, especially weight-bearing sport-specific activity, is positively associated with femoral neck bone density after adjustment for age, sex, ethnicity, smoking, menopausal status, lean body mass, and total body fat [32]. Even after adjusting for age and physical activity, the association of ghrelin with bone density indices remained significant.

Alcohol consumption in adolescence can also cause reduction in bone density [33]. In our study, almost 77% of the volunteers reported they consume alcohol (irrespective of the dosage of the alcohol they drink). Therefore alcohol consumption was adjusted in the analyses as well, and it did not affect the significant results.

Previous studies on the effect of menopause on ghrelin are contradictory. In a study on 57 females, the level of ghrelin was lower in peri-menopausal and post-menopausal women compared to pre-menopausal group, and ghrelin seemed to be positively correlated with bone density [34]. However, another study did not reveal any difference between ghrelin levels of pre- and post-menopausal women [35]. To eliminate the potential influence of menopausal status, the females were divided into two groups based on the menopausal status. We found positive associations between ghrelin and femoral neck, and total hip Z-scores in pre-menopausal women. In post-menopausal group, ghrelin was associated positively with L2-L4 BMD and Z-score. Although this difference might be consequence of changes in sex hormones caused by menopause, factors such as physical activity, body composition, and the smaller number of women in post-menopausal group might also be the reason for the difference observed between these groups.

In the present study, fasting PYY was not significantly associated with bone density. Previous studies evaluated the association between PYY and bone density in metabolic diseases that affect PYY such as anorexia nervosa or in special groups such as athletes. In two studies on anorexia nervosa patients, PYY was negatively associated with bone density [19,36]. However, these studies were done in patients that had lower body weight and usually lower BMD because of the anorexia nervosa [37]. Considering many important factors including smoking, alcohol consumption, and physical activity, it would be difficult to interpret the effect of PYY on bone density in this special group and in small study.

In another study on 47 adolescent girls (aged 12–18 years) in 3 groups of amenorrheic athletes, eumenorrheic athletes, and non-athletic controls, PYY was a negative predictor of lumbar Z-score. In their study, although they controlled for lean mass, other confounding factors such as physical activity, alcohol consumption, and smoking were not entered in the regression model [20]. The amount of physical activity and also age may exert significant influence on BMD and appetite, which in turn could affect PYY.

We did not find any significant association between PYY and bone density indices. Our data suggest that PYY is not likely an important player in determining BMD. The effect of PYY on bone density, which was reported in previous studies, might be through Y2 receptors to reduce NPY [5]. Also, effect of PYY on bone density might be secondary to its effects on body composition and BMI.

Our study had certain limitations. We performed a cross-sectional study and correlation data collected do not prove causality. Therefore, interventional studies of ghrelin administration in osteoporotic patients might be necessary to further evaluate this finding. Also, vitamin D and bone density markers were not measured in our study. Another limitation of our study was that the volunteer-based participation of the subjects in our study resulted in the recruitment of volunteers, where the number of males was less than females. Despite these limitations we are confident that considering the effect of two important gut hormones simultaneously with controlling most of the confounding factors in a big population based study made our results unique and reliable.

## Conclusions

The present study investigated the relationship of two gut hormones, ghrelin and PYY, with BMD in the Newfoundland population. To our knowledge, this is the first study that simultaneously investigates the association of ghrelin and PYY with BMD. It is also the largest population based study adjusting for the most confounding factors in the analysis. With such a large sample size, the present study had significantly higher power than all reported studies to detect the potential statistical signals. The significant positive associations of circulating ghrelin with BMD in women suggest that high levels of ghrelin might have beneficial effects on bone density in the female population. The beneficial effect is independent of BMI, physical activity, age, smoking, and alcohol consumption. The clinical significance of ghrelin on BMD warranted future studies. In our study, PYY was not a significant player in determining bone density.

## Competing interests

The authors declare that they have no competing interests.

## Authors’ contributions

The authors’ roles were as follows; GS and PA: study design, acquisition of data, data analysis, interpretation of data and the writing of the manuscript; FC, YJ, YY, SV: data analysis; PA, FC, DW, PP, YY, HZ, WG, AR, GP: data collection and revision of the manuscript. GS: approval of final version of the manuscript. All authors read and approved the final manuscript.

## Pre-publication history

The pre-publication history for this paper can be accessed here:

http://www.biomedcentral.com/1472-6823/13/35/prepub
